# Potential clinical value of PET/CT in predicting occult nodal metastasis in T1-T2N0M0 lung cancer patients staged by PET/CT

**DOI:** 10.18632/oncotarget.19535

**Published:** 2017-07-25

**Authors:** Xiang Zhou, Ruohua Chen, Gang Huang, Jianjun Liu

**Affiliations:** ^1^ Department of Nuclear Medicine, Ren Ji Hospital, School of Medicine, Shanghai Jiao Tong University, Shanghai, China

**Keywords:** ^18^F-FDG-PET, lung cancer, nodal metastasis, SUVmax

## Abstract

We assessed the clinical value of 2-fluoro-2-deoxyglucose (^18^F-FDG) PET/CT imaging for predicting occult nodal metastasis in non-small cell lung cancer (NSCLC) patients. This retrospective study included 54 patients with T1-2N0M0 NSCLC who had undergone ^18^F-FDG PET/CT before surgery. Occult nodal metastasis was detected in 25.9% (14/54) of the patients. Immunohistochemical analysis revealed that increased glucose transporter 1 expression was associated with occult nodal metastasis, but hexokinase 2 expression was not. Compared to the negative nodal metastasis group, the positive nodal metastasis group was associated with increased maximum standardized uptake value (SUVmax) and tumor size. Multivariate analysis indicated that SUVmax and tumor size were associated with nodal metastasis. Nodal metastasis could be predicted with a sensitivity of 92.9% and a specificity of 55.0% when the SUVmax cutoff was 4.35. When patients were divided into low-risk (tumor size ≤ 2.5 cm and SUVmax ≤ 4.35), moderate-risk (tumor size ≤ 2.5 cm and SUVmax > 4.35 or tumor size > 2.5 cm and SUVmax ≤ 4.35) and high-risk (tumor size > 2.5 cm and SUVmax > 4.35) groups, the lymph node metastasis rates were 4.3%, 22.7%, and 88.9%, respectively. These results indicate that the combination of SUVmax and tumor size has potential clinical value for predicting occult nodal metastasis in NSCLC patients.

## INTRODUCTION

Lung cancer is the leading cause of cancer deaths worldwide in both men and women. Non-small cell lung cancer (NSCLC) accounts for 80%–85% of all cases of lung cancers [1]. The main treatments for patients with stage I NSCLC, especially with lymph node metastasis, are anatomical resection and lymphadenectomy. In patients without lymph node metastasis, limited surgery or stereotactic ablative radiotherapy are possible alternatives [2]. Thus, accurate assessment of lymph node metastasis (N stage) is critical in lung cancer, as it determines the type of surgery.

Positron emission tomography/computed tomography (PET-CT) is a noninvasive molecular imaging tool. PET-CT imaging with the glucose analogue 2-fluoro-2-deoxyglucose (^18^F-FDG) takes advantage of the high glucose metabolism of lung cancer cells and metastatic lesions to visualize not only tumors but also local lymph node metastasis and other distant metastases. Thus, PET-CT has become an important tool for TNM staging of lung cancers [3–6]. Previous studies have demonstrated that PET is more accurate than CT in the detection of lymph node involvement in lung cancer. The predictive ability of intravenous contrast-enhanced CT for mediastinal lymph node metastasis is well documented, and this technique has a sensitivity and specificity of 57%–68% and 76%–82%, respectively. In comparison, integrated ^18^F-FDG-PET/CT is a much better predictor of nodal disease, with a sensitivity of 79–85% and a specificity of 87–92% [7, 8]. However, despite these advantages, the rate of false-positive and false-negative results with PET remains an issue that needs to be resolved. Therefore, there is an urgent need to evaluate the occult nodal metastasis in clinical N0 patients staged by PET/CT based on pathological gold standard to identify the potential occult nodal metastasis predictors.

To predict nodal metastasis and guide clinical treatment in NSCLC patients, it is necessary to investigate the relationship between maximum standardized uptake value (SUVmax) and occult nodal metastasis. In this study, we retrospectively assessed 54 patients with T1-T2N0M0 lung cancer staged by ^18^F-FDG PET/CT before surgery, and analyzed the relationship between PET/CT parameters and occult nodal metastasis after surgery.

## RESULTS

### Clinical characteristics of the patients

Of the 54 lung cancer patients, 34 were men and 20 were women; their average age was 59.25 ± 9.43 years (range, 31–77 years). The average solid tumor size was 1.85 ± 0.91 cm and the mean SUVmax of the primary tumor was 6.29 ± 3.99. The clinicopathological characteristics of the patients are presented in Table 1.

**Table 1:** Baseline characteristics of the study population (n = 54)

Characteristic	n	Percentage
**Age (years)**		
<60	25	46.30%
≥60	29	53.70%
**Gender**		
Male	34	62.96%
Female	20	37.04%
**Tumor size (cm)**		
≤2.5	44	81.48%
>2.5	10	18.52%
**Pathological tumor type**		
Adenocarcinoma	37	68.52%
Squamous cell carcinoma	17	31.48%
**Tumor differentiation**		
Well	31	57.41%
Poor	23	42.59%
**Lymph node metastases**		
Present	14	25.93%
Absent	40	74.07%
**Tumor SUVmax**		
≤4.35	23	42.59%
>4.35	31	57.41%

### The incidence of occult nodal metastasis

N1 involvement was identified in 16.7% (9/54) of the patients, N2 involvement was identified in 9.2% (5/54) of the patients. Thus, occult nodal metastasis was detected in 25.9% (14/54) of the patients. Negative predictive value for occult nodal metastasis on PET/CT was 74.1%.

### The relationship between occult nodal metastasis and clinical features of the patients

**Figure 1:**
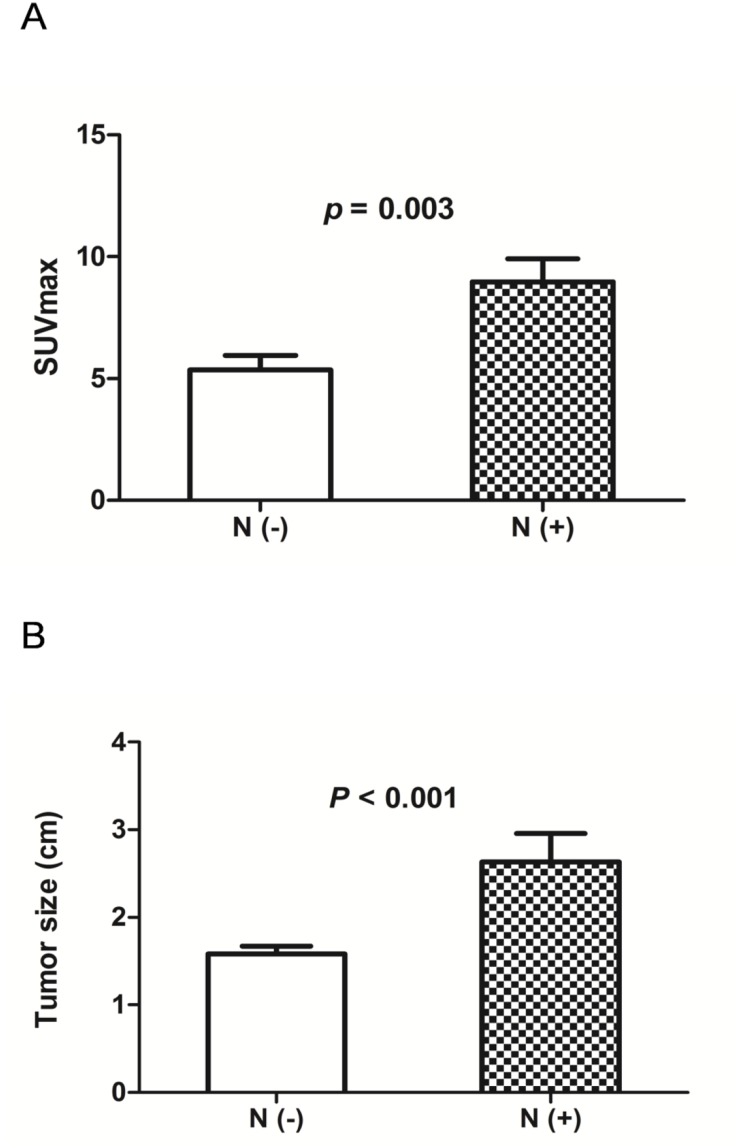
(A) Analysis of SUVmax according to the status of occult nodal metastasis SUVmax was significantly higher in patients with positive nodal metastasis than in those with negative nodal metastasis (P = 0.003). N (+), positive nodal metastasis; N (-), negative nodal metastasis. **(B)** Analysis of tumor size according to the status of occult nodal metastasis. Tumor size was significantly larger in patients with positive nodal metastasis than in those with negative nodal metastasis (P < 0.001). N (+), positive nodal metastasis; N (-), negative nodal metastasis.

**Figure 2:**
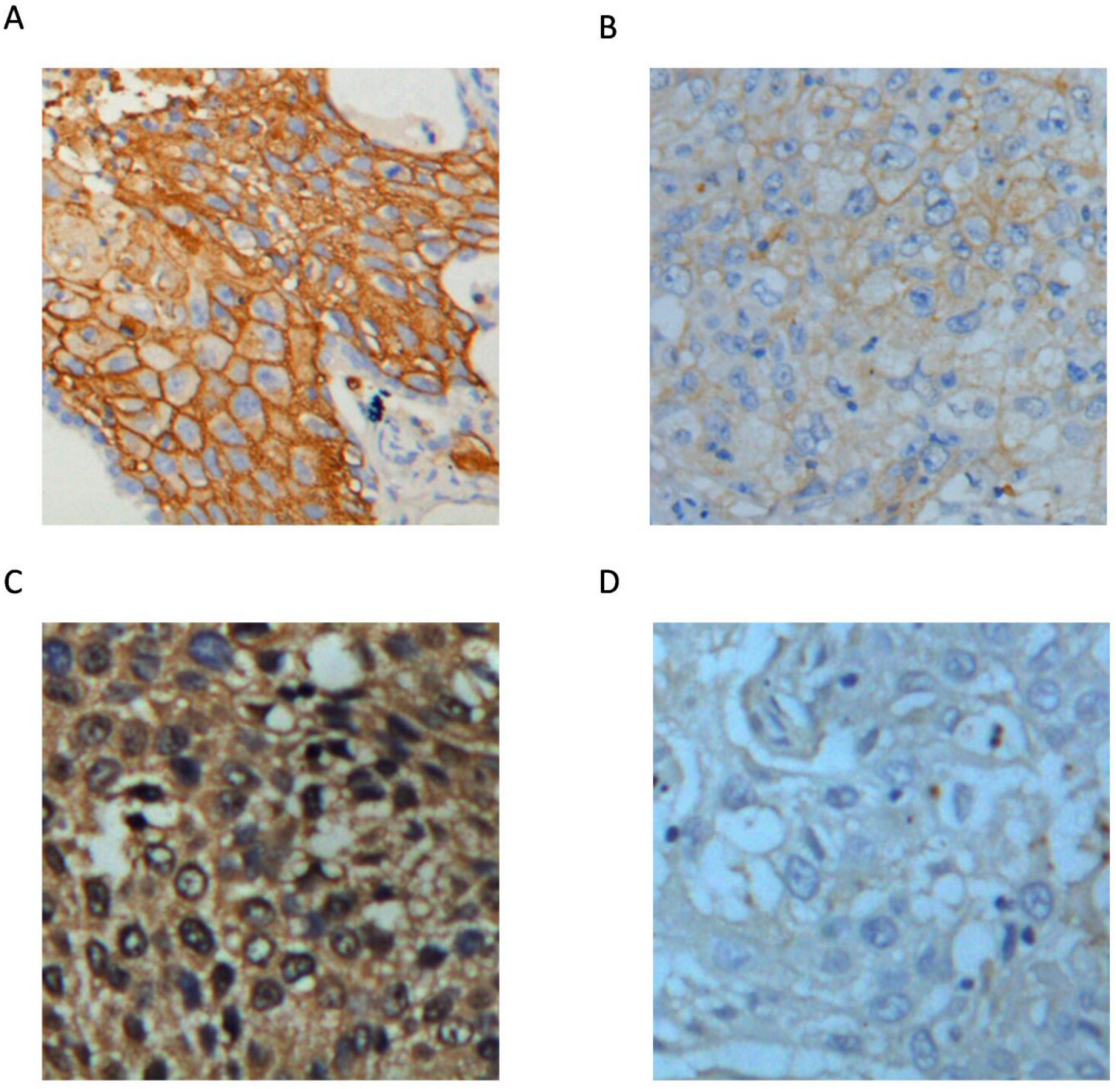
Representative images of GLUT1 and HK2 expression in lung cancer tissues (400×) **(A)** High GLUT1 expression. **(B)** Low GLUT1 expression. **(C)** High HK2 expression. **(D)** Low HK2 expression.

Correlation analysis between occult nodal metastasis and the clinical characteristics of the 54 NSCLC patients was carried out (Table 2). The results showed that occult nodal metastasis did not correlate with age, gender, pathological type, or tumor differentiation. However, occult nodal metastasis was associated with SUVmax of the primary tumor and solid tumor size. Both SUVmax (8.971 ± 0.952, 5.353 ± 0.590, respectively; p = 0.003; Figure 1A) and tumor size (2.632 ± 0.328, 1.580 ± 0.916, respectively; p < 0.001; Figure 1B) were significantly higher in the positive nodal metastasis group compared with the negative nodal metastasis group. SUVmax reflects the glycolytic ability of the tumor. Therefore, we investigated whether the key glycolytic enzymes, glucose transporter 1 (GLUT1) and hexokinase 2 (HK2) were involved in occult nodal metastasis. IHC analysis revealed that GLUT1 expression was associated with occult nodal metastasis (p = 0.043)), but HK2 expression did not correlate with occult nodal metastasis (Table 2 and Figure 2).

**Table 2:** Association between clinicopathological characteristics and lymph node involvement

Characteristics	Nodal status		*p* value
	N(-) (%)	N(+) (%)	
All patients	74.1	25.9	
Age (y)			0.619
<60	76	24	
> 60	70	30	
Gender			0.446
Male	71	29	
Female	80	20	
Pathology			0.692
Squamous cell carcinoma	71	29	
Adenocarcinoma	76	24	
Tumor differentiation			0.201
Well	81	19	
Poor	65	35	
Tumor size			<0.001
≤2.5	86	14	
>2.5	20	80	
Tumor SUVmax			0.002
≤4.35	96	4	
>4.35	58	42	
GLUT-1			0.043
Low	86	14	
High	62	38	
HK-2			0.962
Low	74	26	
High	74	26	

N (+), positive nodal metastasis; N (-), negative nodal metastasis.

### Prediction of occult nodal metastasis and prognosis with ^18^F-FDG uptake values

**Figure 3:**
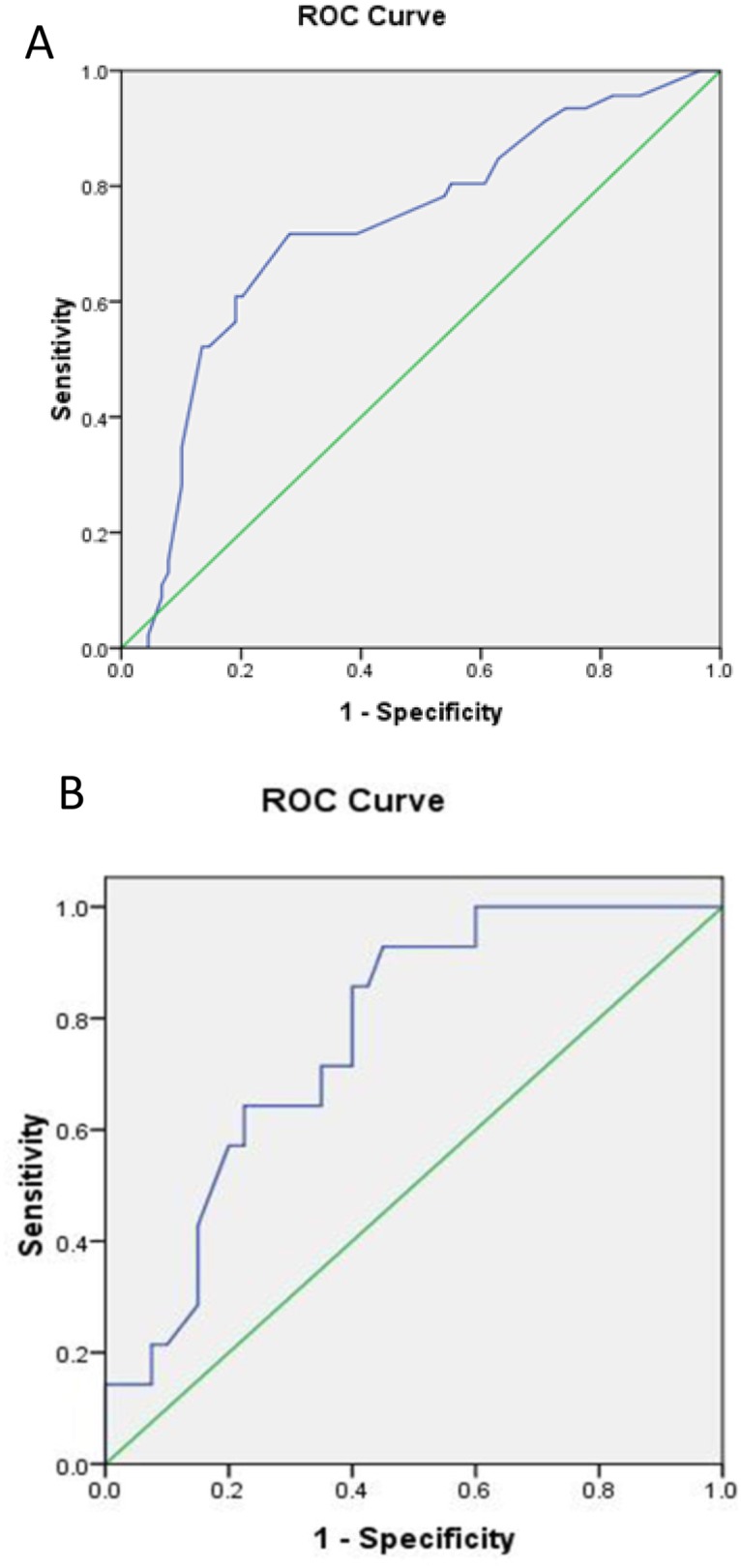
Receiver operator characteristic curve analysis (ROC) of SUVmax for predicting occult nodal metastasis in early lung cancer patients **(A)** ROC of tumor size. **(B)** ROC of SUVmax.

Receiver operating characteristic curve analysis (ROC) was used to determine whether solid tumor size could be used to predict occult nodal metastasis. The results showed that the area under the curve was 0.724 ± 0.047 (Figure 3A). With an optimal cutoff value of 2.5 cm, sensitivity and specificity for the prediction of occult nodal metastasis were 71.7% and 71.9%, respectively.

We further used ROC to determine whether SUVmax could be used to predict occult nodal metastasis. The results showed that the area under the curve was 0.767 ± 0.066 (Figure 3B). With an optimal cutoff value of 4.35, sensitivity and specificity for the prediction of occult nodal metastasis were 92.9% and 55.0%, respectively.

**Figure 4:**
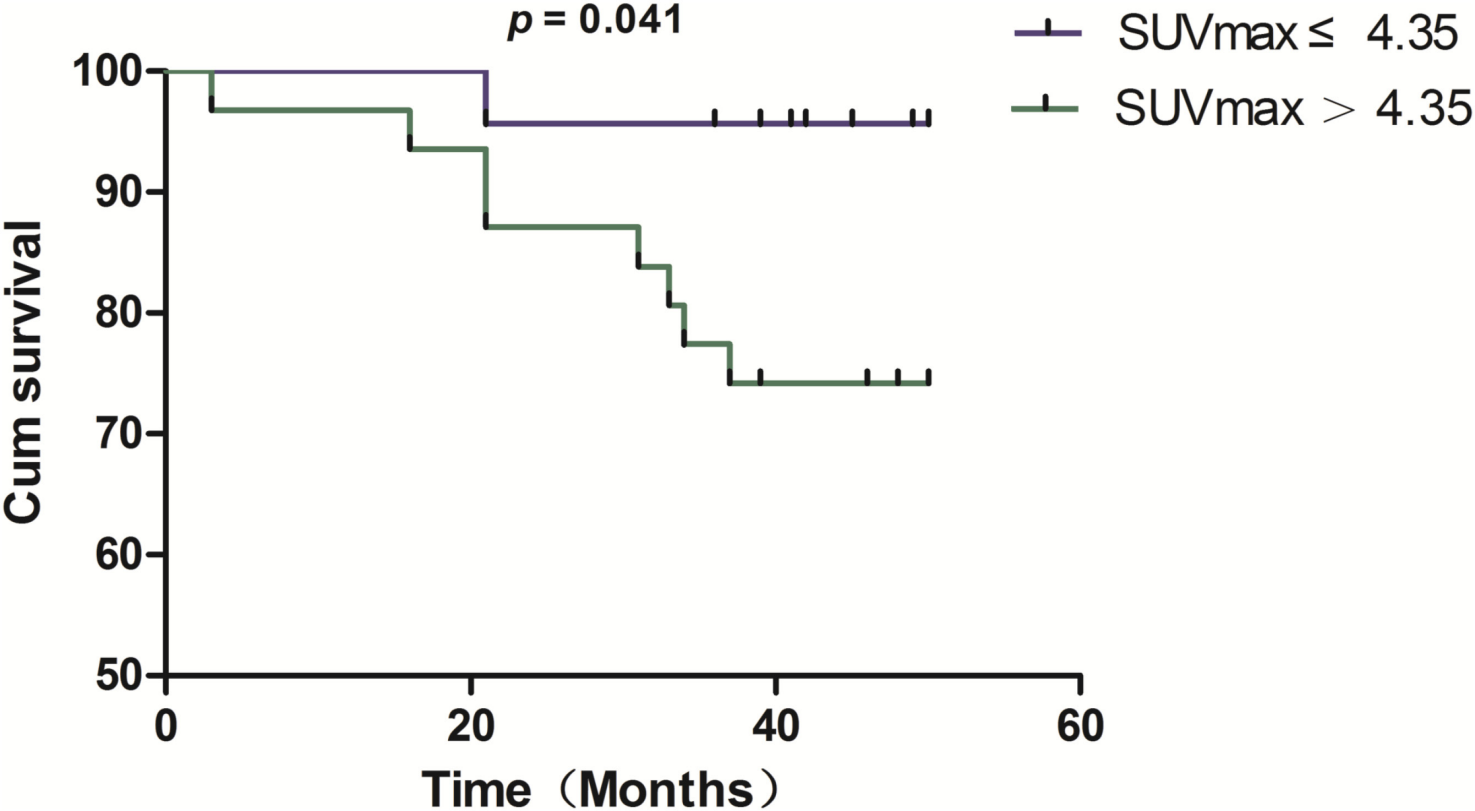
Kaplan–Meier survival analysis reveals a significant difference in overall survival between NSCLC patients with SUVmax ≤ 4.35 and those with SUVmax > 4.35 (p = 0.041)

To investigate the relationship between SUVmax of the primary lesion and prognosis, we divided the patients into two groups by using a cutoff value of 4.35. Of the 23 patients with SUVmax ≤ 4.35, 22 (95.7%) survived and 1 (4.3%) died. Of the 31 patients with SUVmax > 4.35, 23 (74.2%) survived and 8 (25.8%) died. K-M survival analysis was applied to assess the survival of 54 NSCLC patients divided into high and low SUVmax groups (Figure 4). The results showed that survival duration was significantly shorter in the high SUVmax group (p = 0.041).

### Risk factors of occult nodal metastasis

Multivariate analysis revealed that both SUVmax of the primary tumor and tumor size were independent predictors for occult nodal metastasis in lung cancer (Table 3).

**Table 3:** Multivariate analyses of predictors of occult nodal metastasis in NSCLC patients

Variable	Odds ratio	Confidence interval (95%)	*p* value
Tumor size	17.644	2.632-118.283	0.003
SUVmax	10.546	1.073-103.612	0.043

Therefore, using the above parameters, we further divided the patients into three groups based on their risk for occult nodal metastasis: low-risk group, tumor size ≤2.5 cm and SUVmax ≤4.35; moderate-risk group, tumor size ≤2.5 cm and SUVmax >4.35, or SUVmax ≤4.35 and tumor size >2.5 cm; and high-risk group, tumor size >2.5 cm and SUVmax >4.35. The probability of occult nodal metastasis in these three groups was 4.3%, 22.7%, and 88.9%, respectively (p < 0.001) (Table 4).

**Table 4:** Rate of occult lymph node metastasis in the low-, moderate-and high-risk groups

Group	N(+) (%)	N(0)(%)	Total (n)	p value
Low risk	4.3	95.7	23	
Moderate risk	22.7	77.3	22	<0.001
High risk	88.9	11.1	9	
Total	25.9	74.1	54	

## DISCUSSION

SUVmax, a semi-quantitative PET index, is a well-known measure of disease activity or tumor aggressiveness [9]. In this study, we performed PET in 54 early lung cancer patients and found that SUVmax was closely related to nodal metastasis. As SUVmax increased, the probability of nodal metastasis in NSCLC also increased. Through ROC curve analysis, we found that nodal metastasis could be predicted with a sensitivity of 92.9% and a specificity of 55.0% when an SUVmax cutoff value of 4.35 was used; these results are consistent with previous studies. Nguyen et al. [10] analyzed 126 cases of NSCLC and found that the SUVmax of the primary lesion was correlated with nodal metastasis. They also found, using ROC curve analysis, that SUVmax > 6 for the primary lesion could effectively identify local nodal metastasis. Lee et al. [11] analyzed 160 cases of T1 stage NSCLC and predicted local nodal metastasis through SUVmax determined using ^18^F-FDG PET/CT. They reported that the sensitivity, specificity and accuracy of ^18^F-FDG PET/CT for lymph node staging were 11.1%, 86.1%, and 81.9%, respectively. The authors believed that SUVmax of the primary lesion was a good way to predict local nodal metastasis.

SUVmax of the primary tumor represents the local glucose uptake ability. As a higher SUVmax predicts an increased likelihood of nodal metastasis, we conclude that intratumoral glucose metabolism affects local nodal metastasis [12, 13]. Therefore, inhibition of tumor glycolysis to reduce its SUVmax may not only help treat the primary lesion, but also inhibit metastasis to the local lymph nodes, thereby prolonging patient survival. A great deal of biological information is contained within the FDG uptake value of the primary tumor in NSCLC. A better understanding of the FDG uptake mechanisms could explain the more aggressive behavior of NSCLC cells [14].

In addition to metabolic activity, other factors, such as pathological tumor type, degree of differentiation, or tumor size, are associated with nodal metastasis in lung cancer [15]. Therefore, we analyzed the effect of these factors on occult nodal metastasis. Our results showed that while occult nodal metastasis was not associated with pathological type, solid tumor size correlated with nodal metastasis (p < 0.01). Thus, our results indicate that both SUVmax and size of the primary tumor are predictors of occult nodal metastasis in T1-2N0M0 lung cancer patients.

Since determination of nodal metastasis in lung cancer patients directly affects patient prognosis and formulation of surgical strategies, we must effectively utilize other related PET-CT parameters to predict with high accuracy nodal metastasis in NSCLC patients. We therefore divided the study patients into three groups, based on their risk for occult lymph node metastasis: high-risk group, moderate-risk group, and low-risk group. We found that occult nodal metastasis was present in 88.9% of the patients in the high-risk group, but only in 4.3% of the patients in the low-risk group. Therefore, we recommend that high-risk patients undergo lobectomy and lymphadenectomy as soon as possible, while for low-risk patients, limited local surgery is a feasible treatment option.

## MATERIALS AND METHODS

### Study population

54 patients (34 men and 20 women; age range, 31–77 y; mean age, 59 y) who were diagnosed as T1-T2N0M0 lung cancer staged by ^18^F-FDG PET/CT were included in this retrospective study. They were confirmed to have NSCLC based on histopathological findings and underwent surgery after ^18^F-FDG PET/CT between December 2006 and December 2009 at Shanghai Jiaotong University affiliated Ren Ji Hospital. Eligibility criteria were: T1-T2 N0M0; patients did not receive chemotherapy/radiotherapy before PET/CT scanning; performed lobectomy and lymph node dissection; complete case records including age, sex, tumor size, pathological type and histologic differentiation were available; tissue specimens for immunohistochemical staining were available. The Human Investigation Ethical Committee of Shanghai Jiao Tong University affiliated Ren Ji Hospital approved this study. All procedures involving human specimens were performed with written informed consent according to the Declaration of Helsinki.

### PET/CT imaging

A dedicated whole-body PET/CT tomography (SIMENS) was used for all PET/CT imaging. Image acquisition was performed with an integrated PET/CT device. Immediately after CT scanning, PET was performed to cover the identical axial field of view. PET-image data sets were reconstructed with segmented correction for attenuation using the CT data. For semi-quantitative analysis of the ^18^F-FDG uptake, irregular regions of interest (ROIs) were placed over the most intense area of ^18^F-FDG accumulation. The maximum SUV (SUVmax) was obtained for the 1-pixel ROI corresponding to the maximum pixel value in the tumor. Lymph node with SUVmax < 2.5 was regarded as being benign (cN0). Two experienced nuclear medicine physicians, blinded to the clinical history, independently evaluated the PET images and reached a consensus on all image results.

### Immunohistochemistry (IHC) staining

IHC analyses were performed on paraffin-embedded lung cancer tissues. After microtome sectioning (4 μm slices), the slides were processed for staining. Primary antibodies against glucose transporter 1 (GLUT1) and hexokinase 2 (HK2) were purchased from Abcam. The slides were scored for intensity of staining (0 to 3) and the percentages of cells with scores of 0 (0%), 1 (1% to 9%), 2 (10% to 49%), and 3 (50% to 100%) were determined [16]. The IHC score (0 to 9) was defined as the product of the intensity and percentage of cells. Protein expression was judged as high when the IHC score was greater than or equal to 4.

### Statistical analysis

Continuous variables were analyzed by the Student’s t test, and the results were expressed as mean ± standard deviation. Dichotomous variables were analyzed by Chi-squared test or Fisher’s exact test. Kaplan-Meier survival analysis was performed to explore the association between survival and SUVmax. Multivariate logistic regression analysis was used to determine the factors associated with nodal metastasis. Two-sided p values of less than 0.05 were considered statistically significant. All analyses were performed using SPSS software, version 16.0 (SPSS Inc, Chicago, IL).

## CONCLUSION

This retrospective study has revealed that NSCLC patients with high SUVmax and large tumor size on PET/CT constitute a high-risk group for occult lymph nodal metastasis. Thus, these two PET/CT parameters may provide useful information about the metastatic ability of NSCLC, and are of potential clinical significance. However, this is a retrospective study and the sample size was relatively small. Further studies are needed to confirm our results and determine whether the combination of SUVmax and tumor size can be used to predict occult lymph nodal metastasis.
